# Beta-Blocker Propranolol Modulates Decision Urgency During Sequential Information Gathering

**DOI:** 10.1523/JNEUROSCI.0192-18.2018

**Published:** 2018-08-08

**Authors:** Tobias U. Hauser, Michael Moutoussis, Nina Purg, Peter Dayan, Raymond J. Dolan

**Affiliations:** ^1^Max Planck University College London Centre for Computational Psychiatry and Ageing Research, London WC1B 5EH, United Kingdom,; ^2^Wellcome Centre for Human Neuroimaging, University College London, London WC1N 3BG, United Kingdom, and; ^3^Gatsby Computational Neuroscience Unit, University College London, London W1T 4JG, United Kingdom

**Keywords:** decision making, information gathering, noradrenaline

## Abstract

Arbitrating between timely choice and extended information gathering is critical for effective decision making. Aberrant information gathering behavior is thought to be a feature of psychiatric disorders such as schizophrenia and obsessive-compulsive disorder, but we know little about the underlying neurocognitive control mechanisms. In a double-blind, placebo-controlled drug study involving 60 healthy human subjects (30 female), we examined the effects of noradrenaline and dopamine antagonism on information gathering during performance of an information sampling task. We show that modulating noradrenaline function with 40 mg of the β-blocker propranolol leads to decreased information gathering behavior. Modulating dopamine function via a single dose of 400 mg of amisulpride revealed some effects that were intermediate between those of propranolol and placebo. Using a Bayesian computational model, we show that sampling behavior is best explained by inclusion of a nonlinear urgency signal that promotes commitment to an early decision. Noradrenaline blockade promotes the expression of this decision-related urgency signal during information gathering. We discuss the findings with respect to psychopathological conditions that are linked to aberrant information gathering.

**SIGNIFICANCE STATEMENT** Knowing when to stop gathering information and commit to a choice option is nontrivial. This is an important element in arbitrating between information gain and energy conservation. In this double-blind, placebo-controlled drug study, we investigated the role of catecholamines noradrenaline and dopamine on sequential information gathering. We found that blockade of noradrenaline led to a decrease in information gathering. Dopamine blockade showed an intermediate, but nonsignificant, effect. Using a Bayesian computational model, we show that this noradrenaline effect is driven by increased decision urgency, a signal that reflects an escalating subjective cost of sampling. The observation that noradrenaline modulates decision urgency suggests new avenues for treating patients that show information gathering deficits.

## Introduction

A precipitous decision without the benefit of sufficient information is often detrimental, especially if the outcome has nontrivial consequences (e.g., buying a house). In contrast, excess information gathering for trivial decisions (e.g., which toothpaste to buy) wastes both time and energy. A dilemma posed by arbitrating between extended information gathering versus more time-efficient, albeit less well informed, choice is often referred to as a speed–accuracy trade-off (Martin and Müller, 1899; Henmon, 1911).

A speed–accuracy tradeoff is well captured in sequential information gathering tasks (Huq et al., 1988; Chamberlain et al., 2007). Several psychiatric disorders, including schizophrenia, are consistently associated with a reduced propensity to gather information, often termed as “jumping to conclusions.” Such behavior is found in patients (Fear and Healy, 1997; Moutoussis et al., 2011; Dudley et al., 2016), but is also a feature of prodromal states (Rausch et al., 2016) and first-degree relatives (Van Dael et al., 2006). This contrasts with patients diagnosed as having obsessive-compulsive disorder (OCD), who tend to gather more information (Volans, 1976; Fear and Healy, 1997; Pélissier and O'Connor, 2002; Voon et al., 2017; Hauser et al., 2017b,c), although not ubiquitously so (Chamberlain et al., 2007; Jacobsen et al., 2012; Grassi et al., 2015).

The neurocognitive control mechanisms that drive these effects are unknown. Using Bayesian computational modeling, we recently showed that one key contributor is a decision urgency signal that promotes timely decisions (Hauser et al., 2017b,c). The neural processes that modulate urgency signals in sequential information gathering remain unknown. In this study, we examine the role of catecholaminergic neuromodulators in information gathering. Dopamine is implicated in the genesis of schizophrenia (Laruelle, 2013), but previous studies found inconsistent results about its role in jumping to conclusions (Menon et al., 2008; So et al., 2010, 2012; Ersche et al., 2011; Andreou et al., 2014, 2015; Ermakova et al., 2014). Other evidence points to a critical role for noradrenaline, including the role in signaling uncertainty (Aston-Jones and Cohen, 2005; Yu and Dayan, 2005; Dayan and Yu, 2006), suggesting this neuromodulator might play a role during information gathering.

Here, we assessed the contributions of both dopamine and noradrenaline to information gathering in 60 healthy subjects. Using a double-blind, placebo-controlled design, we examined the effects of catecholaminergic antagonists with a high specificity for either dopamine (amisulpride) or noradrenaline (propranolol). We show that blocking noradrenaline by means of propranolol modulates information gathering in an information sampling task, whereas dopamine antagonism by means of amisulpride did not cause a significant change in behavior. Using computational modeling, we demonstrate that the effect of propranolol is best accounted for via alteration of an urgency signal.

## Materials and Methods

### 

#### 

##### Subjects.

We conducted a double-blind, placebo-controlled, between-subjects study involving three groups of 20 subjects each. Subjects were randomly allocated to one of the three groups (ensuring an equal gender balance) after excluding those who met the following exclusion criteria: a history of psychiatric or neurological disorder, regular medication (except contraceptives), current health issues, or any drug allergies. The groups did not differ in their mood (PANAS; Watson et al., 1988), intellectual ability (WASI abbreviated version; Wechsler, 1999), or age (Table 1). Data from the same sample have been reported previously (Hauser et al., 2017a) but addressed a different topic. The University College London research ethics committee approved this study and all subjects provided written informed consent.

**Table 1.** Drug group characteristics

	Placebo	Propranolol	Amisulpride	
Gender (M/F)	10/10	10/10	10/10	
IQ	112.45 ± 12.22	118.75 ± 8.55	114.60 ± 11.77	*F*_(2,57)_ = 1.70, *p* = 0.191
Age	24.50 ± 4.16	23.15 ± 4.31	22.35 ± 2.21	*F*_(2,57)_ = 1.74, *p* = 0.185
Positive affect	29.22 ± 10.47	27.15 ± 7.75	27.80 ± 8.12	*F*_(2,57)_ = 0.286, *p* = 0.752
Negative affect	11.45 ± 2.37	11.95 ± 4.87	11.25 ± 1.92	*F*_(2,57)_ = 0.236, *p* = 0.790

Drug groups were matched for an equal gender balance and did not differ in their age, intellectual abilities (WASI), or affect (PANAS). Data are shown as mean ± SD.

##### Drug groups.

To assess the effects of neurotransmitters dopamine and noradrenaline on information gathering, we used three different drug conditions. The noradrenaline group received 40 mg of propranolol (β-adrenoceptor antagonist). The dopamine group received 400 mg of the D2/3 antagonist amisulpride. We selected these drugs because they have an affinity for the targeted neurotransmitter with a high specificity. The dopamine group received the active drug 120 min before the task and an additional placebo 30 min after the first drug. The noradrenaline group first received a placebo and, after 30 min, the active drug. A third placebo group received placebo at both time points. This schedule aligned with procedures used in previous studies investigating the effects of dopamine or noradrenaline on cognition (Silver et al., 2004; Gibbs et al., 2007; De Martino et al., 2008; Hauser et al., 2017a; Kahnt and Tobler, 2017).

##### Experimental design: information-gathering task.

**Figure 1.**
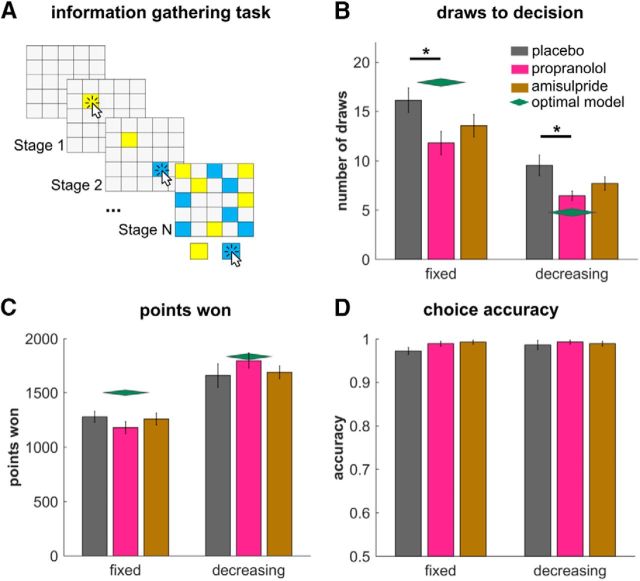
Noradrenaline blockade diminished information gathering. ***A***, Starting from a fully covered deck of cards, subjects were allowed to open as many cards until they felt “certain enough” to indicate whether they believed the majority of the 25 cards was yellow or blue. In a “fixed” condition, no external costs for sampling applied, but in the “decreasing” condition, a potential win of 250 points reduced by 10 points per uncovered card. ***B***, Information gathering is decreased consequent on noradrenaline blockade (propranolol), but not consequent on a dopamine perturbation (amisulpride). This increase in impulsivity is consistently observed across both conditions, rendering the statistics of choices in the noradrenaline group closer to those of an optimal model (green diamonds) in the decreasing condition but further away in the fixed condition. The drug manipulation did not lead to a statistically significant difference in points earned (***C***) and did not affect choice accuracy (***D***; the probability of choosing the color that currently forms the majority of cards at time of decision). *p < 0.05. Data are shown as mean ± SEM.

We examined sequential information gathering using a modified version of an information sampling task (Clark et al., 2006; Hauser et al., 2017b,c). In each game, subjects saw 25 covered cards (gray squares in Fig. 1*A*) and had to decide whether the majority of cards comprised yellow or blue (colors varied across games). Using a computer mouse, the subjects were allowed to open as many cards as they wished before committing to one of the two colors.

The first 15 games belonged to a “fixed” condition in which there was no external cost for sampling more information. Subjects received 100 points for correct decisions and lost 100 points for incorrect decisions independent of the number of cards opened or the time spent on task before decision. The second 15 games belonged to a “decreasing” condition in which information gathering was accompanied by a reduction in potential wins: starting from a potential win of 250 points, opening each card led to a 10-point reduction in wins (e.g., win after 5 opened cards: 250 − 5 * 10 = 200 points). Incorrect decisions were punished with −100 points regardless of the amount of prior sampling.

Before the first game, subjects performed a single practice game to familiarize themselves with the task. After each decision, subjects were informed about their winnings and then directly moved to the next game. The game sequences were selected so that 10 games in each condition were relatively difficult with a generative probability close to 50% (similar to that in the original information sampling task; Clark et al., 2006). An additional 5 sequences were easier with a clearer majority (generative probabilities of a binomial process *p* ∼ 0.7) to allow for a broader variability in information gathering (order of sequences was randomized).

##### Statistical analysis.

In this study, we tested whether blockade of dopamine or noradrenaline function affected information gathering behavior. The number of draws before a decision is a good indicator for the amount of information that a subject opts to collect before making a decision. We thus analyzed this behavioral metric using repeated-measures ANOVA with the between-subject factor group (propranolol, amisulpride, placebo) and the within-subject factor condition (fixed, decreasing). Effects were further assessed using independent-samples *t* tests. Effects sizes are indicated using partial η-squared and Cohen's *d*. As secondary measures, we also assessed whether a group won more points or was less accurate in their decision making (i.e., how often subjects decided for a current minority of cards) using the same statistical procedures.

##### Computational modeling.

To understand cognitive mechanisms of how decisions arise and to probe deeper into how drugs affect these cognitive processes, we used a Bayesian computational model that we previously developed and validated for this task (Hauser et al., 2017b,c). In brief, the winning model assumes that at each state of the game subjects arbitrate between three actions: deciding for yellow, deciding for blue, or continuing with sampling (nondeciding). This arbitration is based on a decision policy, which in turn is based state action Q-values (Watkins, 1989) of each option. The Q-values for deciding in favor of either color are computed as the outcome of a correct/incorrect decision weighted by their inferred likelihood (i.e., “how likely am I to win if I decide for yellow now, given the cards I have opened so far?”). The action value for continuing sampling indexes the value of future states (using backwards induction), plus a subjective cost per step. The latter captures an urgency to decide that arises as sampling continues.

Here, we reiterate the key equations of the winning model for completeness. Please see Hauser et al. (2017c) for a more detailed description and discussion (also cf. Moutoussis et al., 2011; Hauser et al., 2017b). Our model assumes that subjects try to infer the color that forms the majority of cards based on the cards seen so far. This means that the subjects infer the probability that the majority of cards belongs to a particular color; for example, yellow (*y*) *P*(*MY*|*n_y_*, *N*), where *MY* depicts a majority of yellow cards, *n*_y_ the number of opened yellow cards, and *N* the total number of opened cards.

*P*(*MY*) is fully determined as soon as 13 or more of the opened cards belong to one color (of a total number of cards: *N*_tot_ = 25). Otherwise, it can be inferred by calculating the probability of the majority of cards being yellow, given a specific generative probability *q* (proportion of yellow and blue cards in the machinery that produces the sequence) weighted by the likelihood of this generative probability based on the currently seen cards as follows:

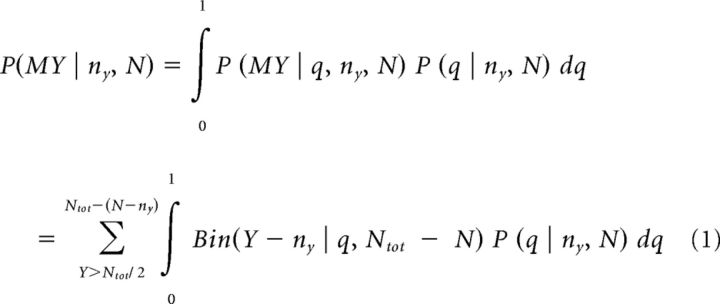
 The first expression is a binomial of getting *Y* − *n*_y_ yellow draws out of *N*_tot_ − *N*, given generative probability *q*. The second expression is the probability of the *q* being the generative probability. This can be calculated using Bayes rule as follows:

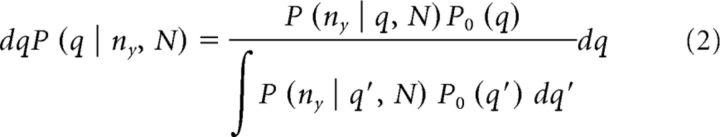
 We assume that the probability of *n* yellow draws of *N* total draws follows a binomial distribution and that the prior belief about the generating *q* follows a beta-distribution with the parameters α and β (here α = 1, β = 1).

The beliefs about the majority of cards are subsequently translated into action values. The action value of choosing *Y*, (*Q(Y*)), is the product of reward/cost of choosing the right or wrong option (*R*_cor_, *R*_inc_) and the success probabilities of these actions. *Q(B*) is calculated analogously as follows:

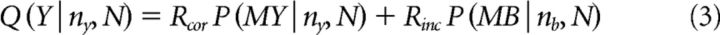
 The rewards of correctly (*R*_cor_) and incorrectly declaring (*R*_inc_) can be cast in different ways. According to the objective instructions, in the fixed condition *R*_cor_ is set to 100 and *R*_inc_ to −100. For the decreasing condition, we compared two different formulations. In our main model (“subjective”), we also kept *R*_cor_ constant in the decreasing condition. This was done so that the subjective costs (*c*_s_, cf. below) soak up the subjectively perceived overall costs; that is, a combination of externally imposed and internally generated costs. This way, we can investigate the subjectively perceived total costs. Alternatively, we formulated an “objective” costs model, where *R*_cor_ changes as a function of step (250, 240, 230, …), as set up in the task. This objective model only differed in the decreasing condition, not in the fixed condition. Additionally, *R*_inc_ was kept at −100 for all models and conditions (as per instructions). For the simulation of an optimal model (green diamonds in Fig. 1), we used the objective model and assumed no additional costs per step.

The action value of not deciding (*Q(ND*)) computes the value of future states in terms of the future action values and their probabilities. Additionally, a cost per step is imposed that assumes that there are internal (and external) costs that emerge when continuing with sampling (urgency signal). *Q(ND*) is calculated using backward induction to solve the Bellman equation, using state values *V(s'*) and a cost per step *c*_s_ as follows:

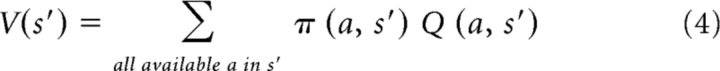


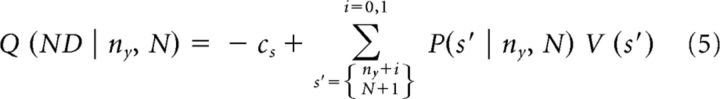
 The probability of reaching state *s'* and seeing *i* new yellow items is based on the current belief state, which in turn is mainly determined by the current evidence *n*_y_, *N*:

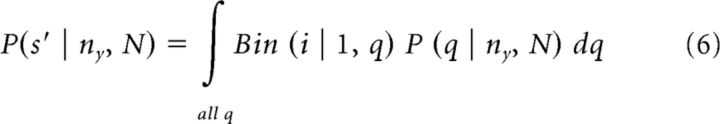
 The choice policy π for the state action space is specified as the following softmax function with decision temperature parameter τ and irreducible noise (lapse rate) parameter ξ (cf. Guitart-Masip et al., 2012) as follows:

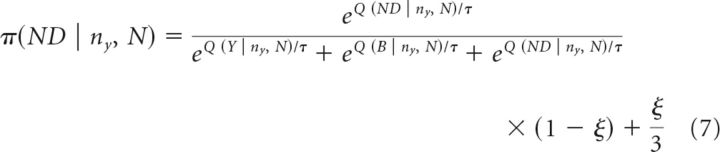


### Decision urgency: nonlinear sigmoidal cost function

We compared two different possible cost functions: fixed cost per sample (“linear”) versus a model in which the costs per sample increased according to a sigmoid function (“nonlinear,” cf. below). The latter could capture the possibility that subjects felt an increasing urgency to decide (Cisek et al., 2009), for instance, if it becomes increasingly annoying to gather more samples with potentially little informational content and waste time, similar to previous reports that show that costs increase nonlinearly (Drugowitsch et al., 2012; Murphy et al., 2016).

We implemented the nonlinear cost function as a sigmoid, where the cost per step *c*_s_ (Eq. 5) changes on each step *n* (1, …, 25) as follows:

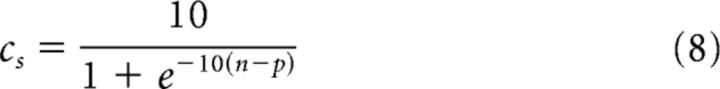
 The free impatience parameter *p* describes the indifference point; that is, at what stage in the game the agent becomes impatient. We set the scaling factor as well as the slope parameter to 10. Model comparison revealed that fixing these parameters led to an equal performance in terms of model fit and thus outperformed other, more complex models that had these parameters as free parameters. Parameter *p* was independently modeled in the two conditions, allowing for different urgency trajectories.

Studies investigating urgency in perceptual decision making have used different forms of nonlinear urgency signals, such as exponentials (Drugowitsch et al., 2012). In our task, these urgency signals are different in that they influence the planning (value of nondeciding) rather than a decision threshold directly. This means that the actual costs for continuing sampling are weighted sums of the future costs and, therefore, even if *c*_s_ itself plateaus, the adversarial effects can still grow (ultimately linearly). We lack sufficient data for determining the exact form of the nonlinearly escalating urgency.

#### 

##### Model fitting and model comparison.

We optimized the parameters to maximize a log likelihood for each participant individually. We used a genetic algorithm implemented in MATLAB (Goldberg, 1989) (300 generations, crossover-heuristic for generation of children, four individuals to survive in next generation). Initial population/parameter for all subjects was based on results from a previous study (Hauser et al., 2017b) and we bounded the parameter space based on these previous studies, their definitions within the model, and their psychological meaning (*p*: [0 25], τ: [1 10], ξ: [0 0.5]). In every case, we ensured the best-fitting parameters each fell within these boundaries.

For model comparison, we used summed Akaike information criterion (AIC; Akaike, 1973) and Bayesian information criterion (BIC; Schwarz, 1978). The best-fitting model was then used for further analyses, as reported in the Results section.

Similar to our previous findings (Hauser et al., 2017b,c), a subjective costs model (AIC: 13079, BIC: 13333) clearly outperformed an objective model that incorporates the explicit external costs (AIC: 17875, BIC: 18129). Further, we found that a model with a nonlinear cost function outperformed a linear cost model (AIC: 9709, BIC: 10133). This means that decision urgency arises in a nonlinear, sigmoidal fashion, escalating as sampling continues. This model also outperformed a logistic regression model recently proposed by Malhotra et al. (2017), where choice was predicted by evidence difference and time (AIC: 17582, BIC: 17920). Therefore, our findings are consistent with the sort of nonlinear urgency-signals considered to influence a class of perceptual decision making problems (Churchland et al., 2008; Cisek et al., 2009; Drugowitsch et al., 2012; Murphy et al., 2016).

**Figure 2.**
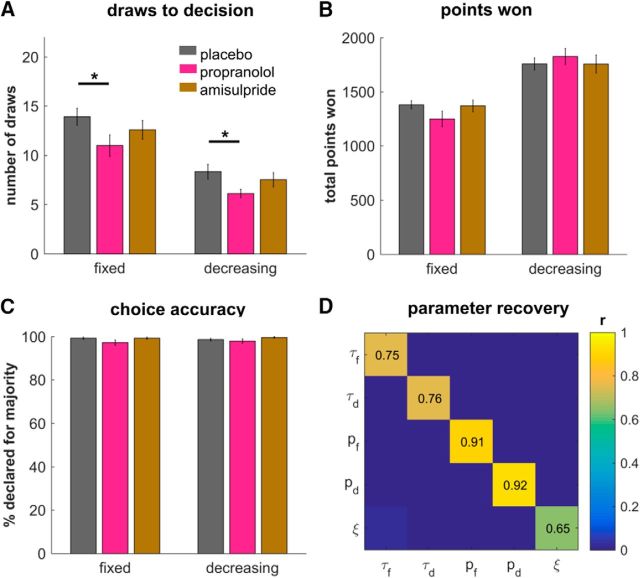
Best-fitting model reproduced behavior. The model with a nonlinear urgency reproduced subjects' observed behavior. We simulated agents with the individual parameters associated with subjects, recovering the differences between placebo and propranolol in the sampling (***A***), and showing similar patterns in performance (***B***) and accuracy (***C***). ***D***, Parameter recovery analyses were reassuring, especially for the urgency parameters *p*. *p < 0.05. Data are shown as mean ± SEM.

Further model comparison revealed that a model with only one free parameter *p* (midpoint of a sigmoid) per condition in the cost function achieved the same model fits as more complex versions with additional free parameters and thus outperformed these models in terms of AIC and BIC (AIC: 9331, BIC: 9585). Simulations using this winning model revealed similar behavioral patterns as seen in subjects' actual behavior (Fig. 2*A–C*). The significant difference for sampling under propranolol, the similar number of draws before decision, and the total points won suggests that our winning model captures subjects' actual task behavior and describes group differences accurately. This means that we can investigate the model to better understand whether there are meaningful computational differences between groups. Moreover, we performed a parameter recovery analysis of the winning model and found that we are well able to recover the parameters (Fig. 2*D*).

To assess how noradrenaline modulated the computational mechanisms underlying information gathering, we focused on a comparison between placebo and propranolol and whether the decision urgency arose at different time points. To do this, we derived the model parameter *p* that modulates the emergence of the urgency signals as well as the model-derived urgency signal itself. A group difference for the latter was assessed using cluster-extent permutation tests (Nichols and Hayasaka, 2003), similar to that used in our previous studies (Hauser et al., 2017b,c). Because of the non-independence between samples, we computed the cluster size of the true effect and compared it against a null distribution of cluster sizes from randomly permuted data (*p* < 0.05, height threshold *t* = 1, 1000 permutations). This is a common approach in neuroimaging data analyses, for which the data show similar features (Hunt et al., 2013; Hauser et al., 2015).

## Results

### Noradrenaline blockade decreases information gathering

An analysis of the number of draws before declaring a choice as an index for information gathering revealed a main effect of group, supporting a significant drug effect on information gathering (Fig. 1*B*, *F*_(2,57)_ = 4.29, *p* = 0.018, η^2^ = 0.131). Follow-up analysis showed that this effect was driven by a reduction in information gathering in the noradrenaline compared with a placebo group in both conditions (fixed condition: *t*_(38)_ = 2.55, *p* = 0.015, *d* = 0.81; decreasing condition: *t*_(38)_ = 2.71, *p* = 0.010, *d* = 0.86). The dopamine group showed an intermediate effect, but did not significantly differ from either group (fixed condition: vs placebo: *t*_(38)_ = 1.53, *p* = 0.134, *d* = 0.48; vs noradrenaline: *t*_(38)_ = 1.08, *p* = 0.286, *d* = 0.34; decreasing condition: vs placebo: *t*_(38)_ = 1.47, *p* = 0.150, *d* = 0.47; vs noradrenaline: *t*_(38)_ = 1.47, *p* = 0.149, *d* = 0.46). A split-half between early and later trials replicated the propranolol effect for each half and did not reveal any additional amisulpride effect, arguing against drug-induced learning differences. A main effect of condition, but no interaction with group, indicated that subjects across placebo and drug conditions gathered more information in the fixed compared with the decreasing condition (condition main effect: *F*_(1,57)_ = 145.92, *p* < 0.001, η^2^ = 0.719; interaction: *F*_(2,57)_ = 0.528, *p* = 0.592, η^2^ = 0.018).

### No significant drug effects on winnings or accuracy

To test whether the noradrenaline or dopamine drug affected other aspects of performance, we compared the total points won as well as accuracy of decisions. There was no group effect on winnings (Fig. 1*C*, *F*_(2,57)_ = 0.04, *p* = 0.961, η^2^ = 0.001) and no interaction (*F*_(2,57)_ = 1.67, *p* = 0.198, η^2^ = 0.055), suggesting that the reduced information gathering in noradrenaline was not large enough to affect subjects' winnings. This absence of a difference in winnings (placebo vs propranolol: fixed condition: *t*_(38)_ = 1.32, *p* = 0.195, *d* = 0.42; decreasing: *t*_(38)_ = −1.06, *p* = 0.294, *d* = 0.33) reflects the fact that most sequences had a generative probability close to 50%. Therefore, a decrease in information/decrease in costs under propranolol was not large enough to make it through to actual winnings. The groups did not differ in choice accuracy (Fig. 1*D*, evident in a lack of main effect of group: *F*_(2,57)_ = 1.90, *p* = 0.159, η^2^ = 0.063; the absence of an interaction: *F*_(2,57)_ = 0.86, *p* = 0.428, η^2^ = 0.029). This indicates the drug manipulation did not affect general motivation to solve the task correctly.

### Propranolol promotes urgency to decide

**Figure 3.**
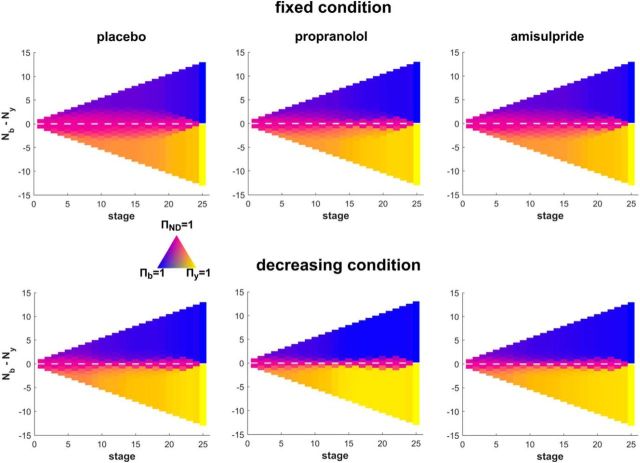
Model policy reflecting the choice probability for choosing yellow, blue, or continuing sampling (pink) depending on the evidence difference (*y*-axis) and the information-gathering stage (*x*-axis). This depiction shows that subjects are less likely to sample in the decreasing condition in general (less pinkish areas). Also, one can clearly see how a nondecision area is diminished in the propranolol group (middle) compared with the placebo group (left).

To understand the cognitive processes that drive a lowered information gathering disposition in the noradrenaline group, we fitted a Bayesian computational model (Hauser et al., 2017b,c) to individual subjects' data. Decision policy of the best fitting model as a function of evidence and sampling is depicted in Figure 3.

**Figure 4.**
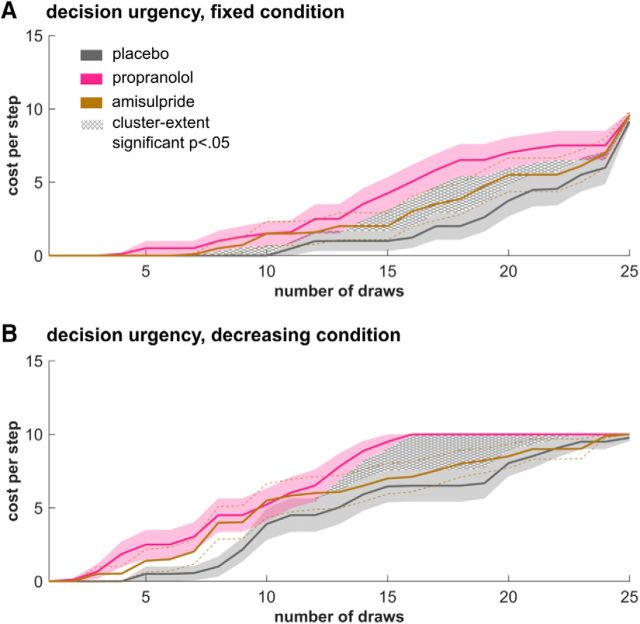
Premature urgency drives noradrenaline-modulated decreases in information gathering. A cost-per-step signal reflects an emerging urgency to commit to a decision as sampling progresses. This urgency signal arises nonlinearly, escalating significantly earlier in the noradrenaline group in the fixed (***A***) and the decreasing (***B***) condition. The group mean of the urgency signal (±1 SEM) is derived from a sigmoidal model function for subjective costs, which outperformed linear models.

In the model, the speed with which subjects make a decision is modulated by two separate processes: a finite horizon and subjective urgency. The former arises because there is a limit on the number of cards such that whenever 13 cards of the same color are opened, opening more cards does not provide further information. The latter, however, is not built into the task explicitly (especially in the fixed condition), but rather is based on a finding that subjects apparently become more liberal in their decision criterion as sampling progresses (Cisek et al., 2009; Drugowitsch et al., 2012; Thura and Cisek, 2014; Murphy et al., 2016; Malhotra et al., 2017). In the model, urgency is captured by a subjective cost for each sample and we found that it arises in a nonlinear fashion (being low in the beginning but escalates over the course of sampling). The cost arose significantly earlier in the noradrenaline compared with placebo condition in both conditions (Fig. 4, fixed: *p* = 0.003, decreasing *p* = 0.029), reflecting their lower number of draws.

We compared the individually fitted model parameters between groups and found a group effect in the repeated-measures ANOVA (*F*_(2,57)_ = 4.42, *p* = 0.016, η^2^ = 0.134) for the parameter *p*. This describes the midpoint of an escalating urgency signal. Subsequent *t* tests revealed that the noradrenaline group expressed significantly lower parameter values in both conditions (Table 2; *p*_fixed_: *t*_(38)_ = 2.60, *p* = 0.013, *d* = 0.82; *p*_decreasing_: *t*_(38)_ = 2.94, *p* = 0.006, *d* = 0.93), meaning that subjective costs/urgency arise significantly earlier in the sampling process. The dopamine group, again, did not differ from other groups (*p*_fixed_: vs placebo: *t*_(38)_ = −1.30, *p* = 0.201, *d* = 0.41; vs noradrenaline: *t*_(38)_ = 1.21, *p* = 0.232, *d* = 0.38; *p*_decreasing_: vs placebo: *t*_(38)_ = −1.10, *p* = 0.279, *d* = 0.35; vs noradrenaline: *t*_(38)_ = 1.54, *p* = 0.132, *d* = 0.48). The choice stochasticity parameter τ (*F*_(2,57)_ = 0.25, *p* = 0.783, η^2^ = 0.009) as well as the lapse rate parameter ξ (*F*_(2,59)_ = 1.10, *p* = 0.340, η^2^ = 0.037) did not yield significant group differences.

**Table 2.** Model parameter comparison

	Placebo	Propranolol	Amisulpride	Placebo vs propranolol
*p*_f_	21.01 ± 4.47	16.52 ± 6.27	18.86 ± 5.88	*t*_(38)_ = 2.60, *p* = 0.013
*p*_d_	13.90 ± 5.89	9.14 ± 4.23	11.77 ± 6.38	*t*_(38)_ = 2.94, *p* = 0.006
τ_f_	2.85 ± 2.23	3.30 ± 2.47	3.26 ± 2.58	*t*_(38)_ = −0.60, *p* = 0.550
τ_d_	5.10 ± 2.74	4.73 ± 2.35	5.52 ± 2.68	*t*_(38)_ = 0.45, *p* = 0.653
ξ	0.004 ± 0.009	0.007 ± 0.008	0.004 ± 0.007	*t*_(38)_ = −1.21, *p* = 0.234

Group comparison reveals that the propranolol group has significantly lower indifference point parameters for both the fixed (*p*_f_) and the decreasing (*p*_d_) condition. This means that their urgency arises earlier in an information-gathering process and thus drives them to make more hasty decisions. None of the other parameters was significantly different. Data are shown as mean ± SD.

A similar effect was found when analyzing a (worse-fitting) logistic regression (as suggested in Malhotra et al., 2017) in which a time-dependent predictor was significantly increased in the propranolol group (fixed condition: placebo β = 0.858 ± 0.576; propranolol: β = 1.189 ± 0.431; *t*_(38)_ = −2.05, *p* = 0.047; decreasing condition: placebo: β = 1.375 ± 0.672; propranolol: β = 1.987 ± 0.914; *t*_(38)_ = −2.41, *p* = 0.021), with no difference in the intercept (fixed condition: placebo β = −3.941 ± 0.962; propranolol: β = −3.561 ± 0.906; *t*_(38)_ = −1.28, *p* = 0.207; decreasing condition: placebo: β = −3.440 ± 1.302; propranolol: β = −3.603 ± 1.337; *t*_(38)_ = 0.39, *p* = 0.698), which hints toward a stronger collapse of a decision threshold rather than a lowered initial threshold. However, we caution that the insights afforded by the simplicity of this analysis does not compensate for its limited accuracy in capturing the data compared with the cognitive model (in terms of fit quality or BIC).

## Discussion

An arbitration between gathering more information and time efficiency is a nontrivial aspect of decision making and it is thought to go awry in a range of psychiatric disorders such as schizophrenia or OCD (Fear and Healy, 1997; Moutoussis et al., 2011; Hauser et al., 2017b,c). We show that inhibiting noradrenergic function by means of a noradrenergic β-adrenoceptor blockade leads to decreased information gathering. Using computational modeling, we show that noradrenaline blockade has a primary effect on a component of the decision process that reflects an “urgency to decide.” Urgency signals facilitate decision making and have primarily been described in perceptual decision making (Cisek et al., 2009; Drugowitsch et al., 2012; Thura and Cisek, 2014; Murphy et al., 2016). Our results demonstrate that a similar urgency signal plays a role in sequential information gathering, in which decision urgency arises nonlinearly.

Our computational modeling showed that noradrenaline directly modulates how quickly an urgency signal arises. Inhibiting noradrenaline function led to an earlier emergence of this urgency signal. Theories of noradrenaline functioning suggest that it influences cognition at different timescales, with tonic and phasic noradrenaline expressing distinct functional roles (Aston-Jones and Cohen, 2005; Yu and Dayan, 2005; Dayan and Yu, 2006). Because our manipulation is likely to influence both tonic and phasic noradrenaline, a specific contribution of one or the other cannot be inferred from our data. We note a recent perceptual decision-making study (Murphy et al., 2016) supports the idea that phasic noradrenaline might be critical for decision urgency. The authors showed that phasic pupil response, a potential indicator for phasic noradrenaline (Joshi et al., 2016), is linked to longer decision times, possibly signaling a delayed emergence of an urgency signal. Whether such phasic noradrenaline promotes urgency directly or if it is mediated indirectly by signaling unexpected uncertainty (Dayan and Yu, 2006) that in turn delays an arising urgency can be addressed in future studies.

Our findings suggest that manipulating noradrenaline could benefit patients whose information gathering is aberrant. In particular, we showed previously that compulsive subjects and patients with OCD gather information excessively and that this is due to a delayed emergence of decision urgency (Hauser et al., 2017b,c). The antagonistic effect of propranolol on this urgency signal raises the theoretical possibility that this agent might alleviate an indecisiveness found in OCD patients. We are not aware of any study that has investigated the role of noradrenergic β-receptor blockade in the treatment of OCD. Indirect evidence for the viability of this mode of intervention comes from observation with the therapeutic agent clomipramine. This is a first-line treatment for OCD (Koran et al., 2007) the mode of action of which comprises complex noradrenergic effects including a downregulation of β-adrenoceptor density (Asakura et al., 1982) that might attenuate the impact of endogenous noradrenaline on β-adrenoceptor related functions. Additionally, augmenting clomipramine with pindolol, a β-blocker, is reported to have positive effects on OCD symptoms (Koran et al., 2007; Sassano-Higgins and Pato, 2015). These findings would support a more systematic examination of whether propranolol has beneficial effects for OCD patients in general or, more specifically, for patients in whom there is evidence for prepotent excessive information-gathering behavior.

Previous studies on the effects of dopaminergic drugs on sequential information gathering have produced mixed results. In schizophrenia, antipsychotic medication does not directly alleviate jumping-to-conclusion behavior (Menon et al., 2008; So et al., 2010, 2012) and even remitted patients express intermediate levels of information gathering (Moutoussis et al., 2011). In healthy subjects, only one study found more cautious decision making after dopamine D2/3 agonist administration (Ersche et al., 2011), whereas several other studies did not find an effect of either dopamine-enhancing or -reducing medications (Andreou et al., 2014, 2015; Ermakova et al., 2014). Using amisulpride, we found no significant effect on information gathering. However, in almost all analyses, the effects of amisulpride were intermediate between placebo and propranolol and were nonsignificantly different from propranolol. These results suggest that amisulpride induces a similar, albeit weaker, effect than our noradrenaline manipulation. Whether this is due to a less direct influence of dopamine on information gathering or if it might be due to a lower effective dose of amisulpride (compared with propranolol) remains unclear. We believe the latter is less likely because there have been several studies showing significant effects of such a dose on cognition (Ramaekers et al., 1999; Kahnt and Tobler, 2017), but multiple administrations of amisulpride, as in patient treatment, might reveal a different effect.

A limitation of this study is that, although we selected drugs that preferentially target dopamine or noradrenaline function, these agents have known effects on other systems. In particular, although propranolol has a high affinity for β-adrenoceptors (Fraundorfer et al., 1994), it also has a lesser affinity for serotonin receptors (Hamon et al., 1990). Moreover, similar to other studies that have investigated the effect of amisulpride on cognitive functioning, we used only a single dose of this agent (Kahnt and Tobler, 2017; Burke et al., 2018). However, this single dose is not comparable to the impact of long-term antipsychotic treatment, in which the therapeutic dosage of amisulpride is often higher and there are likely to be complex effects on dopamine transmission. In addition, to be comparable to the clinical studies, we decided to use a relatively brief task. Although the length of this task provides consistent behavioral findings regarding when subjects decide on average, a more extended task would allow us also to compute other measures such as behavioral decision thresholds. Future, more extended studies could provide additional insight about when and how an urgency signal affects a decision threshold and whether it purely affects the collapse or also its plateau.

In conclusion, we show that information gathering is modulated by noradrenergic β-adrenoceptor blockade. Our computational modeling shows this is mediated via a modulation of a subjective, nonlinear urgency-to-respond signal. The effect of dopaminergic receptor blockade remained inconclusive, with a much weaker effect than that seen for propranolol. An involvement of noradrenaline in this aspect of decision making opens potential avenues for therapy in psychiatric conditions in which there is aberrant information gathering.
